# Predicting Postoperative Recurrence Using a Support Vector Machine for Patients With Esophageal Squamous Cell Carcinoma: Machine Learning Modeling Development and Validation Study

**DOI:** 10.2196/68027

**Published:** 2025-10-23

**Authors:** Meng Qing Xu, Zhi Sheng Jiang, Wan Yu Liao, Ying Kang, Xiao Yue Feng, Kang Jiang, Qiong Jiang, Zhuang Zhuang Cong, Jing Luo, Lin Wu, Yi Shen, Fang Yu Wang

**Affiliations:** 1 Department of Gastroenterology, Jinling Hospital, Affiliated Hospital of Medical School, Nanjing University, No. 305, Zhongshan East Road, Nanjing, 210002, China, 86 17826080919; 2 Department of Cardiothoracic Surgery, Jinling Hospital, Affiliated Hospital of Medical School, Nanjing University, Nanjing, China; 3 Department of Gastroenterology, Jinling Hospital, Jinling School of Clinical Medicine, Nanjing Medical University, Nanjing, China

**Keywords:** esophageal squamous cell carcinoma, ESCC, nomogram, risk factors, SVM, support vector machine, postoperative recurrence, esophageal cancer, morbidity, mortality rates, surgery, adjuvant chemotherapy, chemotherapy, oncology, malignancy, artificial intelligence, AI

## Abstract

**Background:**

While numerous models have been developed to predict overall survival in postoperative patients with esophageal squamous cell carcinoma (ESCC), few have specifically focused on predicting postoperative recurrence.

**Objective:**

This study aimed to develop and validate a support vector machine (SVM)-based predictive model for evaluating recurrence risk and identifying associated factors in ESCC patients following surgery.

**Methods:**

We retrospectively analyzed clinical data from 311 ESCC patients who underwent surgery at Jinling Hospital between June 2014 and November 2016, with follow-up until October 2021 (median of 36 follow-up months, range 0-93.5 months). After excluding cases with incomplete data (n=1), 310 eligible patients were randomly allocated into test (n=106), validation 1 (n=103), and validation 2 (n=101) cohorts. Using SVM algorithms, patients were stratified into high- or low-recurrence-risk groups. Model performance was assessed using sensitivity, specificity, the Youden index, positive predictive value, and negative predictive value. Calibration curves were generated to evaluate model accuracy and reliability. Statistical analyses were performed using SPSS (version 22.0; IBM Corp) and R (version 3.6.1; R Foundation for Statistical Computing).

**Results:**

In all cohorts, SVM7 (incorporating tumor node metastasis [TNM] stage, adjuvant therapy, differentiation, tumor size, and complications) demonstrated significantly higher sensitivity in predicting recurrence than SVM6 (based on the Eastern Cooperative Oncology Group performance status, neutrophil-to-lymphocyte ratio, and CY211) (*P*<.001). The composite model SVM6+8 (combining SVM6 and SVM8 [SVM7 excluding complications]) achieved recurrence prediction sensitivities of 94%, 79.59%, and 72.73% in the test, validation 1, and validation 2 groups, respectively; with specificities of 98.11%, 69.84%, and 78.43%. These results were comparable to SVM6+TNM (SVM6 combined with TNM staging) but outperformed SVM6 alone (*P*<.001). Survival analysis revealed significantly longer disease-free survival in the SVM6+TNM-predicted low-risk group compared to the high-risk group, with a marked difference in recurrence rates (*P*<.001).

**Conclusions:**

The proposed SVM-based model enables accurate prediction of postoperative recurrence in ESCC patients with high sensitivity, specificity, and discriminative power, offering a valuable tool for clinical risk stratification.

## Introduction

Esophageal cancer poses a threat to public health due to its high morbidity and mortality rates [1,2]. Postoperative tumor node metastasis (TNM) staging is the most valuable index for evaluating the prognosis of patients with esophageal squamous cell carcinoma (ESCC). However, it is an index that can only be confirmed after surgery and can only provide a theoretical basis for postoperative treatment strategies. Thus, it is of little significance for the planning of surgical strategies before surgery in individual patients, especially for those with a poor physical condition or in whom performing surgery is difficult, as it is difficult to evaluate the TNM staging of these patients.

Numerous factors affect the postoperative recurrence rate in patients with ESCC [3], including postoperative complications, Eastern Cooperative Oncology Group (ECOG) performance status, clinicopathological characteristics, tumor markers, and inflammatory as well as nutritional indicators. These inflammatory and nutritional indicators include the neutrophil-to-lymphocyte ratio (NLR), C-reactive protein-to-prealbumin ratio (CPR) [4], platelet×C-reactive protein multiplier (P-CRP), lymphocyte-to-monocyte ratio, Glasgow prognostic score (GPS), and other inflammatory markers [5], all of which affect postoperative survival and prognosis. Tumor marker levels are widely recognized for their prognostic value in predicting postoperative recurrence of esophageal cancer [6,7]. However, studies that include other clinical indicators are relatively limited. A comprehensive and systematic analysis of preoperative blood indicators, patient-specific conditions, intraoperative factors such as duration of surgery and blood loss, and postoperative complications is necessary to identify key risk factors. Screening these indicators and constructing an optimal predictive model will have significant clinical value in improving postoperative management and patient outcomes.

Recently, an increasing number of studies have focused on predictive models. Here, we present the development and validation of a clinical prediction model using a support vector machine (SVM). The SVM was used to develop a more robust model for predicting postoperative recurrence compared with other approaches. As a new data mining methodology, SVM has been applied to predict tumor progression and clinical outcomes by integrating molecular markers and clinical features [8-10]. Furthermore, this method is suitable for small patient cohorts, where independent and random assignment into 3 groups enhances the reliability of analysis and validation. Given its advantages, SVM is likely to continue to provide valuable insights into the accurate prediction of the recurrence of ESCC [11,12]. We collected information on commonly used clinical blood indicators and surgical data, and the patients were followed up to analyze potential risk factors. Through iterative combinations of these factors weighted by their relative importance, we developed an optimal recurrence prediction model. Our goal is to integrate the indices from the optimal SVM model into an artificial intelligence model for patients with ESCC who have not yet had an individualized treatment plan developed.

## Methods

### Patients and Follow-Up

Baseline data was obtained from the medical records of patients diagnosed with ESCC between June 2014 and November 2016 at Jinling Hospital. Data was abstracted in December 2016 by 2 independent researchers (MQX and ZSJ) and a study database created, of which basic information was used for follow-up. These patients were followed up until October 2021. The collected data primarily comprised preoperative information, including basic information, blood indicators (inflammation, infection, and tumor markers), presence or absence of adjuvant therapy, intraoperative blood loss, duration of surgery, and postoperative complications. Follow-ups were conducted approximately every 3 months, primarily through phone calls. If the patients could not be reached, we obtained their contact details from the outpatient department, and additional attempts were made to establish communication. When phone contact was unsuccessful, we sent letters to or conducted home visits. Patients who remained unreachable were considered lost to follow-up and were excluded from the study.

### Inclusion and Exclusion Criteria

We included patients who met the following criteria: (1) patients had a diagnosis of ESCC confirmed by a postoperative histopathological examination, (2) they had radical resection for ESCC, (3) they had complete clinical and follow-up data, and (4) the surgery was performed by the same surgeon. Patients who met the following criteria were excluded: (1) they had liver or kidney dysfunction or hematological disease; (2) they had a concurrent or previous history of other malignant tumors; (3) they had perioperative death, defined as mortality due to serious complications within 1 month postoperatively; and (4) they were receiving preoperative chemoradiotherapy.

### Statistical Analysis

Data were analyzed using SPSS (version 22.0; IBM Corp) and R (version 3.6.1; R Foundation for Statistical Computing [13]). Univariate and multivariate analyses of the relative prognostic importance of parameters were performed using the Cox proportional hazards model. An SVM uses implicit mapping of input data into a high-dimensional feature space using a kernel function [14]. Learning occurs in this feature space based on the “kernel trick.” Due to its popularity in machine learning and pattern classification, numerous SVM packages are available, such as *LIBSVM* and *KERNLAB*. In this study, we used the R package *KERNLAB*. The SVM model was developed using perioperative data, inflammation markers, and tumor markers to predict ESCC recurrence. From SVM model 1 (SVM1) to SVM6, in the initial analysis, we evaluated all potential predictors through correlation and Cox proportional hazards regression. Candidate variables showing statistically significant associations with esophageal cancer recurrence (*P*<.05) underwent receiver operating characteristic (ROC) curve evaluation. All risk factors were area under the curve (AUC)-ranked and iteratively pruned to optimize the SVM model’s predictor set. An identical approach was applied from SVM7 to SVM10. SVM1 included all preoperative markers (ECOG, NLR, CPR, CY211, squamous cell carcinoma antigen [SCC], P-CRP, GPS, and age); SVM2 included factors in SVM1 excluding P-CRP; SVM3 included factors in SVM2 excluding GPS; SVM4 included factors in SVM3 excluding SCC; SVM5 included factors in SVM4 excluding age; SVM6 included factors in SVM5 excluding CPR (final variables: ECOG, NLR, and CY211); SVM7 included TNM, adjuvant therapy, differentiation, tumor size, and complications; SVM8 included factors in SVM7 excluding complications; SVM9 included factors in SVM8 excluding tumor size; and SVM10 included factors in SVM9 excluding differentiation. ROC curve analysis was performed for each SVM model, and the AUC values were used to calculate the predictive ability of the SVM models for recurrence.

All patients included in the study were randomly assigned to the test, validation 1 (Val1), or validation 2 (Val2) groups. Using the SVM algorithm, each group was further assigned to a high- or low-risk of recurrence group. In the test group, we combined several predictive indicators of recurrence to stratify patients into high- and low-risk subgroups. The predictive performance of this integrated predictive model was then validated in 2 independent cohorts (Val1 and Val2). The Kaplan-Meier method was used to calculate and plot recurrence curves, further validating the ability of the SVM models to distinguish patients with a high and low risk of recurrence. Sensitivity, specificity, the Youden index, the positive predictive value (PPV), and the negative predictive value (NPV) were assessed to evaluate the practical value of the model. *χ*^2^ tests were used to analyze differences in sensitivity, specificity, PPV, and NPV among the SVM models. A calibration curve was created using the Hosmer-Lemeshow goodness-of-fit test to assess the degree of calibration of the model to ensure its accuracy and reliability. All tests were 2-sided, and *P*<.05 was considered statistically significant.

### Ethical Considerations

This study was approved by the Institutional Ethics Review Board (IERB 2018NZKY-021‐03) of the Ethics Committee of Jinling Hospital. Verbal informed consent was obtained by telephone during follow-up communications. Standard university hospital guidelines, in accordance with the principles detailed in the Declaration of Helsinki, were followed in handling patient tissues and publication, ensuring confidentiality and anonymity. All participants who completed the survey received a complimentary disease knowledge resource as a token of appreciation and compensation for their participation.

## Results

### Basic Patient Information

We collected data from 311 patients with postoperative ESCC, which included 241 men (77.5%) and 70 women (22.5%) with a median age of 66 years (range 40-83 y). Preoperative data, blood indicators, intraoperative blood loss, duration of surgery, TNM stage, degree of differentiation, postoperative adjuvant therapy, and complications are shown in Multimedia Appendix 1. The results of quantitative correlation analysis between preoperative tumor markers and postoperative clinical indicators are shown in Multimedia Appendix 2. Postoperative complications included pulmonary infection, incision infection, gastrointestinal dysfunction, recurrent nerve injury, severe pulmonary infection, respiratory failure, hydropneumothorax, anastomotic fistula, anastomotic or thoracic fistula, and hemorrhage requiring re-operation. On October 15, 2021, 144 (46.3%) patients were recurrence-free, whereas 167 (53.7%) had a recurrence. The postoperative follow-up period ranged from zero to 93.5 months (median 36 mo), concluding in October 2021. The postoperative disease-free survival (DFS) was 78.7% at 1 year, 59% at 3 years, and 53.6% at 5 years (see Multimedia Appendix 3).

### Risk Factors for Recurrence and Predictive Ability

According to univariate and multivariate Cox regression model analyses, age, ECOG performance status, NLR, CPR, CY211, TNM staging, and postoperative complications were identified as independent risk factors (see Tables1  2). Postoperative adjuvant therapy and ECOG performance status showed the highest predictive ability, as measured using the AUC values (AUC=0.63, 95% CI 0.570-0.695), followed by NLR (AUC=0.599, 95% CI 0.536-0.663). The predictive ability of CY211, CPR, tumor size, and cell differentiation was lower than that of TNM staging (AUC=0.676, 95% CI 0.615-0.737; see Table 3).

**Table 1.** Risk factors affecting the recurrence of patients with esophageal squamous cell carcinoma (ESCC) by Cox single factor analysis.

Clinical parameters	B	SE	Wald	*df*	Univariate	*P* value
					HR^a^ (95% CI)	
Age (years)	−0.335	0.168	3.993	1	0.715 (0.515-0.994)	.05
Gender	−0.168	0.203	0.689	1	0.845 (0.568-1.258)	.41
ECOG^b^	1.162	0.183	40.492	1	3.198 (2.235-4.574)	<.001
NLR^c^	0.69	0.171	16.395	1	1.995 (1.428-2.786)	<.001
LMR^d^	−0.214	0.167	1.634	1	0.808 (0.582-1.121)	.20
P-CRP^e^	0.342	0.168	4.151	1	1.407 (1.013-1.955)	.04
GPS^f^	0.394	0.168	5.511	1	1.483 (1.067-2.062)	.02
CRP^g^ (mg/dL)	0.242	0.167	2.101	1	1.274 (0.918-1.769)	.15
CPR^h^	0.592	0.17	12.176	1	1.808 (1.297-2.523)	<.001
SCC^i^ (ng/ml)	0.419	0.17	6.064	1	1.521 (1.089-2.122)	.01
CY211(ng/ml)	0.651	0.172	14.388	1	1.918 (1.370-2.685)	<.001
Surgical method	−0.12	0.176	0.465	1	0.887 (0.628-1.253)	.50
Tumor location	0.078	0.163	0.229	1	1.081 (0.785-1.490)	.63
Intraoperative blood loss	0.228	0.191	1.423	1	1.256 (0.864-1.828)	.23
Operative time	−0.106	0.168	0.394	1	0.900 (0.647-1.251)	.53
Tumor size	0.611	0.189	10.485	1	1.843 (1.273-2.669)	.001
T^j^	0.666	0.173	14.894	1	1.947 (1.388-2.731)	<.001
N^k^	1.407	0.176	63.598	1	4.085 (2.890-5.773)	<.001
TNM^l^	1.337	0.174	59.174	1	3.807 (2.708-5.351)	<.001
Cell differentiation	0.815	0.174	21.909	1	2.258 (1.606-3.176)	<.001
Adjuvant therapy	0.867	0.171	25.835	1	2.380 (1.704-3.325)	<.001
Complications	0.464	0.171	7.401	1	1.591 (1.139-2.222)	.007

a HR: hazard ratio.

b ECOG: Eastern Cooperative Oncology Group.

c NLR: neutrophil-to-lymphocyte ratio.

d LMR: lymphocyte to monocyte ratio.

e P-CRP: platelet × C-reactive protein multiplier.

f GPS: Glasgow prognostic score.

g CRP: C-reactive protein.

h CPR: C-reactive protein-to-prealbumin.

i SCC: squamous cell carcinoma antigen.

j T: size or extent of the primary tumor.

k N: regional lymph nodes.

l TNM: tumor node metastasis.

**Table 2.** Risk factors affecting the recurrence of patients with esophageal squamous cell carcinoma (ESCC) by Cox multiple factor regression analysis.

Clinical markers	B	SE	Wald	*df*	Multivariate (HR^a^ (95% CI)	*P* value
Age (≥66 vs <66 years)	−0.552	0.174	10.03	1	0.576 (0.409-0.810)	.002
ECOG^b^ (≥1 vs <1)	1.362	0.199	46.682	1	3.905 (2.642-5.772)	<.001
NLR^c^ (≥2.43 vs <2.43)	0.553	0.183	9.118	1	1.739 (1.214-2.489)	.003
CPR^d^	0.539	0.18	9.001	1	1.714 (1.206-2.438)	.003
CY211 (≥2.65 vs <2.65 ng/mL)	0.526	0.178	8.777	1	1.692 (1.195-2.396)	.003
TNM^e^ (III+IV vs I+II）	1.389	0.18	59.238	1	4.010 (2.816-5.712)	<.001
Complications	0.533	0.182	8.571	1	1.704 (1.193-2.435)	.003

a HR: hazard ratio.

b ECOG: Eastern Cooperative Oncology Group.

c NLR: neutrophil-to-lymphocyte ratio.

d CPR: C-reactive protein-to-prealbumin.

e TNM: tumor node metastasis.

**Table 3.** The area under the curve (AUC) of receiver operating characteristic (ROC) curves with preoperative and postoperative clinical markers in predicting postoperative recurrence in patients with esophageal squamous cell carcinoma (ESCC).

Clinical indexes	AUC^a^ (95% CI)		*P* value
Reference	0.500 (—)		—^b^
Age (years)	0.445 (0.381-0.510)		.10
ECOG^c^	0.633 (0.571-0.695)		<.001
NLR^d^	0.599 (0.536-0.663)		.003
P-CRP^e^	0.539 (0.475-0.604)		.23
GPS^f^	0.543 (0.478-0.607)		.20
CPR^g^	0.591 (0.527-0.654)		.006
SCC^h^ (ng/ml)	0.555 (0.491-0.619)		.09
CY211(ng/ml)	0.598 (0.534-0.661)		.003
Tumor size	0.573 (0.510-0.637)		.03
TNM^i^	0.676 (0.615-0.737)		<.001
Cell differentiation	0.571 (0.507-0.635)		.03
Adjuvant therapy	0.633 (0.570-0.695)		<.001
Complications	0.550 (0.486-0.614)		.13

a AUC: area under the curve.

b Not applicable.

c ECOG: Eastern Cooperative Oncology Group.

d NLR: neutrophil-to-lymphocyte ratio.

e P-CRP: platelet × C-reactive protein multiplier.

f GPS: Glasgow prognostic score.

g CPR: C-reactive protein-to-prealbumin.

h SCC: squamous cell carcinoma antigen.

i TNM: tumor node metastasis.

### SVM Combined With the ROC Model for Predicting Recurrence

The SVM model combined with ROC analysis was used to predict recurrence. In the test, Val1 and Val2 groups, the sensitivity of SVM2—which included all preoperative markers—for predicting recurrence was 94.12%, 70.59%, and 60.98%, respectively, with a specificity of 98.21%, 63.33%, and 56.86%, respectively. The sensitivity of SVM6—which included ECOG, NLR, CY211—in the test, Val1, and Val2 groups was 67.86%, 60.47%, and 68.18%, respectively, with a specificity of 86%, 63.33%, and 64.91%, respectively. The sensitivity of SVM7—which included TNM, adjuvant therapy, differentiation, tumor size, and complications—in the test, Val1, and Val2 groups was 92.86%, 76.74%, and 84.09%, respectively, with a specificity of 76%, 61.67%, and 71.93%, respectively (see Multimedia Appendix 4). No significant difference was observed between the sensitivity and specificity of SVM2 and SVM7 (*P*>.05). However, SVM6 had a lower sensitivity for predicting recurrence than SVM7. The sensitivity of SVM6+8 for predicting recurrence was 94%, 79.59%, and 72.73% in the test, Val1, and Val2 groups, respectively, with a specificity of 98.11%, 69.84%, and 78.43%, respectively. These sensitivities were comparable with those of SVM6+TNM, and the specificities were higher than those of SVM6+TNM (*P*<.001; see Table 4).

**Table 4.** Comparison among different marker combinations obtained before surgery and after surgery and all markers according to sensitivity, specificity, positive predictive value (PPV), negative predictive value (NPV), and accuracy in predicting patients’ recurrence.

Variable combinations	Test + validation group 1 + validation group 2 (n=310)														
	Sensitivity, %	*χ* ^2^	*P* value^a^	Specificity, %	*χ* ^2^	*P* value	PPV^b^, %	*χ* ^2^	*P* value	NPV^c^, )	*χ* ^2^	*P* value	Accuracy, %	*χ* ^2^	*P* value
SVM^d^ 2	76.22	3.804^e^	.05	73.05	0.526^e^	.468	70.78	0.003^e^	.96	78.21	2.000^e^	.16	74.52	0.429^e^	.51
		3.819^f^	.05		0.237^f^	.63		0.872^f^	.35		2.406^f^	.12		2.854^f^	.09
SVM 6	65.73	14.830^e^	<.001	70.66	0.057^e^	.81	65.73	0.829^e^	.36	70.66	8.338^e^	.004	68.38	5.479^e^	.02
SVM 7	85.31^e^	—^g^	—	69.46^e^	—	—	70.52^e^	—	—	84.67^e^	—	—	76.77^e^	—	—
SVM 6+8	82.52	0.414^e^	.52	81.44	6.465^e^	.01	79.19	3.174^e^	.08	84.47	0.002^e^	.96	81.93	2.520^e^	.11
		0.414^h^	.52		3.339^h^	.07		1.626^h^	.20		0.042^h^	.84		1.02^h^	.31
SVM 6+9	85.31	<0.001^e^	≥.99	73.05	0.526^e^	.47	73.05	0.269^e^	.60	85.31	0.023^e^	.88	78.71	0.336^e^	.56
		0.901^i^	.34		0.060^i^	.81		0.146^i^	.70		0.711^i^	.40		0.590^i^	.44
SVM 6+ TNM^j^	81.12	0.901^e^	.34	71.86	0.231^e^	.63	71.17	0.017^e^	.90	81.63	0.466^e^	.50	76.13	0.036^e^	.86
		0.094^k^	.76		4.282^k^	.04		2.676^k^	.10		0.441^k^	.51		3.154^k^	.07

a *P* value: Corresponding comparisons.

b PPV: positive predictive value.

c NPV: negative predictive value.

d SVM: support vector machine.

e χ2 test was used for comparisons among markers obtained SVM 2, SVM 6, and SVM 7, and among markers of SVM6+8, SVM6+9, SVM 6+TNM, and SVM7, respectively.

f χ2 test was used in comparisons between markers of SVM 2 and SVM 6.

g Not available.

h *χ*2 test was used for comparisons between markers of SVM 6+8 and SVM 6+9.

i *χ*2 test was used in comparisons between markers of SVM6+TNM and SVM 6+9.

j TNM: tumor node metastasis.

k *χ*2 test was used in comparisons between markers of SVM 6+8 and SVM6+TNM.

### Multifactor Integrated Analysis for the Prediction of Postoperative DFS

**Figure 1.**
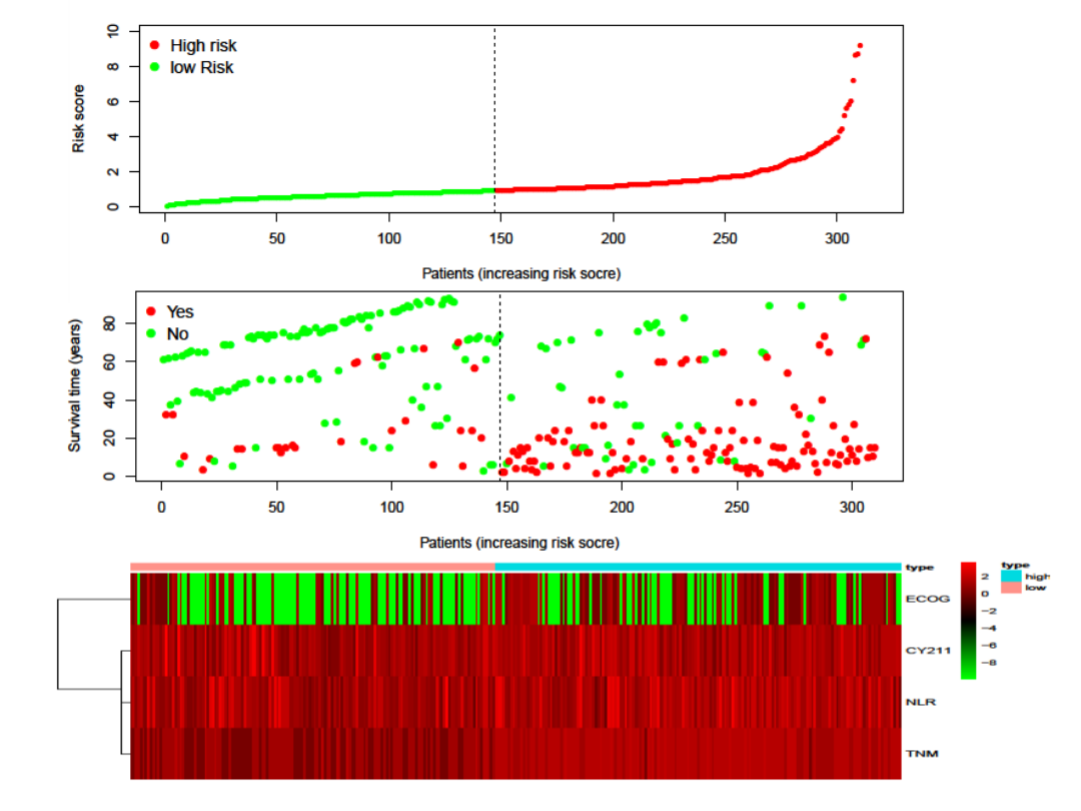
Heatmap of high and low distribution profiles of risk factors affecting the recurrence of patients with esophageal squamous cell carcinoma (ESCC). ECOG: Eastern Cooperative Oncology Group; NLR: neutrophil-to-lymphocyte ratio; TNM: tumor node metastasis.

**Figure 2.**
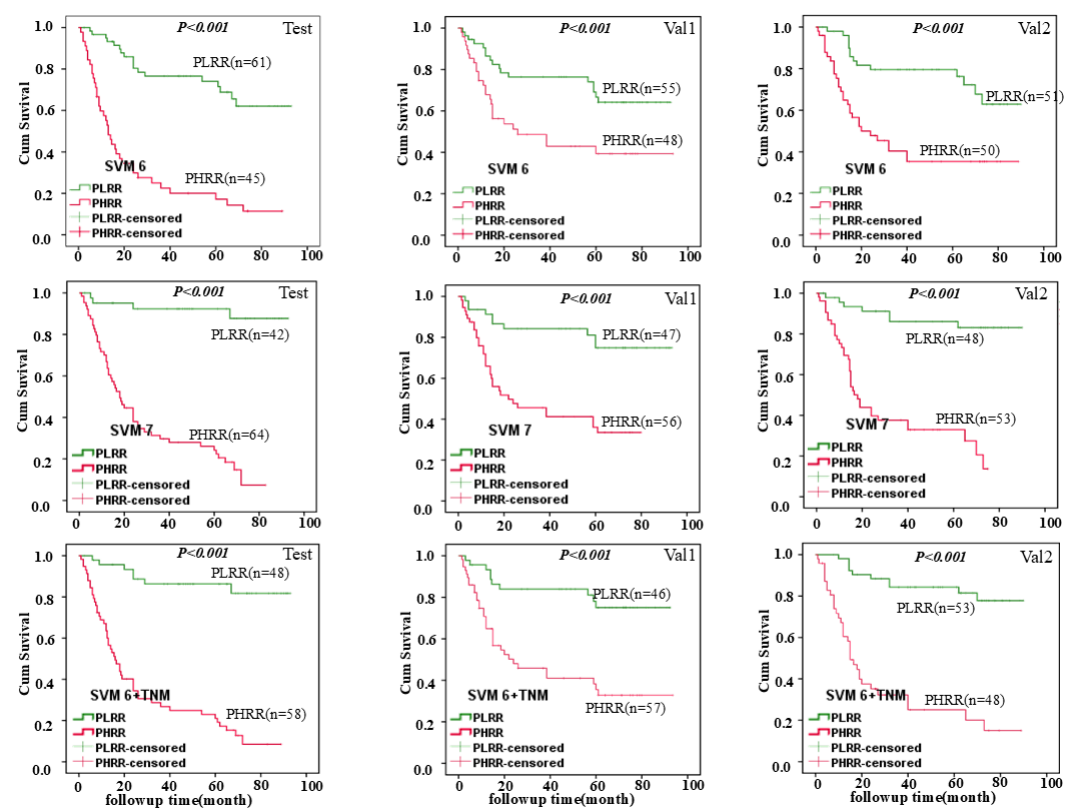
Survival analyses were performed for the low-risk versus high-risk groups of SVM6, SVM7, and SVM6+TNM models. We randomly divided the 311 patients into test, Val1, and Val2 groups, and then each group was divided into a high-risk recurrence group and a low-risk recurrence group. Kaplan-Meier survival analysis showed that the average postoperative survival time of patients with esophageal squamous cell carcinoma (ESCC) in the low-risk recurrence group was longer than that of the high-risk group among test, Val1, and Val2 groups (SVM6, SVM7, and SVM6+TNM models). (*P*<.001). SVM: support vector machine; PLRR: predicted low-recurrence-risk; PHRR: predicted high-recurrence-risk; TNM: tumor node metastasis.

We generated a heatmap showing the high- and low-distribution profiles of risk factors affecting recurrence in patients with ESCC (see Figure 1 and Multimedia Appendix 5). Postoperative survival analysis revealed that the DFS of the predicted low recurrence risk group in the SVM6 and SVM6+TNM models was much longer than that of the predicted high recurrence risk group. A considerable difference in cumulative survival rates was also observed (see Figure 2).

### Development and Validation of a Nomogram for the Prediction of DFS

**Figure 3.**
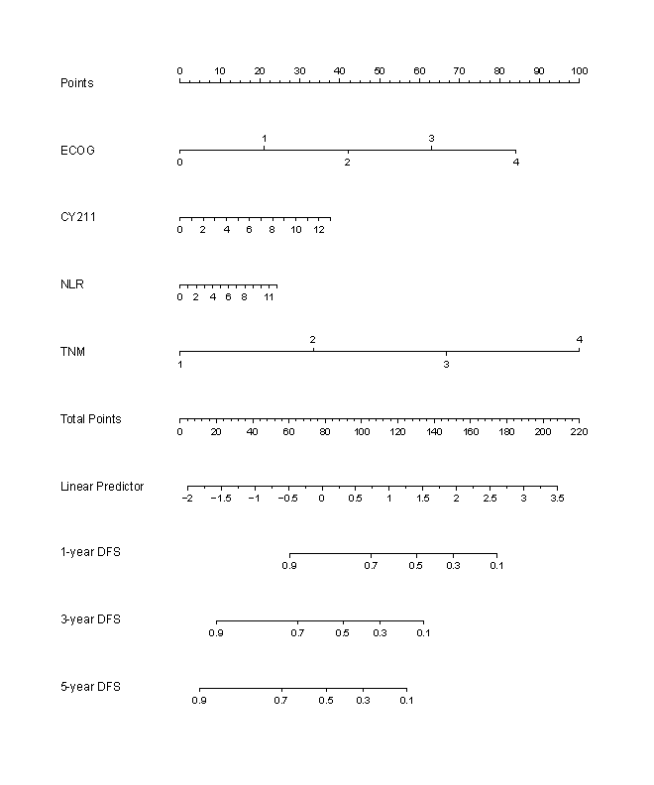
The nomogram (SVM 6+TNM) predicted individual patient-level 1-, 3-, and 5-year disease-free survival (DFS) based on preoperative and postoperative clinical index. Vertical lines were drawn from the correct status of each prognostic factor to the top axis (points). After the addition of all the points, a vertical line was drawn from the “total points” axis to the bottom axes. This helps in the conversion into a 1-, 3-, and 5-year DFS probability. ECOG: Eastern Cooperative Oncology Group; NLR: neutrophil-to-lymphocyte ratio; TNM: tumor node metastasis; DFS: disease-free survival.

**Figure 4.**
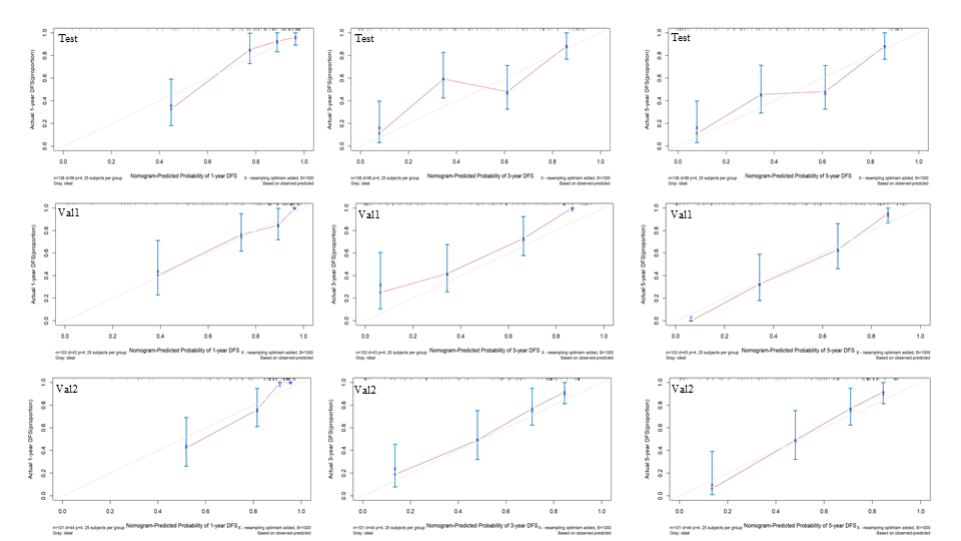
Calibration curve of 1-, 3-, and 5-year disease-free survival (DFS) in the training set and internal validation set. The error bars represent the 95% CI of these estimates. Val: validation.

A nomogram was developed using the available data to predict DFS. Vertical lines were drawn from the correct status of each prognostic factor on the top axis (points). Summing all points allowed for the projection of a vertical line from the “total points” axis to the bottom axes, facilitating the conversion into 1-, 3-, and 5-year DFS rates (see Figure 3 and Multimedia Appendix 6). The SVM6-based nomogram demonstrated reliable performance in predicting DFS, with an AUC of 0.769. Postoperative outcomes were predicted and evaluated with a sensitivity of 65.73%, specificity of 70.66%, and PPV of 83.54%. Similarly, the SVM6+TNM-based nomogram effectively predicted DFS with an AUC of 0.847 (see Table 5), offering sufficient sensitivity (81.12%) and specificity (71.86%) for postoperative assessment. This nomogram provides valuable insights for guiding treatment decisions and follow-up plans in patients with ESCC. The calibration curves were used to evaluate the consistency of the nomogram (SVM6+TNM and SVM 6). The findings indicated a high degree of uniformity between the predicted and observed probabilities of survival in the training set and internal validation set (see Figure 4 and Multimedia Appendix 7).

**Table 5.** Receiver operating characteristic curves for the support vector machines (SVMs) models using testing data, validation 1 data, and validation 2 data separately.

Combinations	Test			Val^a^ 1			Val 2			Val 1+2		
	AUC^b^ (95% CI)		*P* value	AUC (95% CI)		*P* value	AUC (95% CI)		*P* value	AUC (95% CI)		*P* value
Before surgery												
SVM^c^ model 1	0.962 (0.919-1.000)		<.001	0.650 (0.547-0.753)		.007	0.579 (0.462-0.697)		.19	0.618 (0.540-0.696)		.004
SVM model 2	0.962 (0.919-1.000)		<.001	0.670 (0.568-0.771)		.002	0.589 (0.472-0.707)		.14	0.633 (0.556-0.710)		.001
SVM model 3	0.916 (0.856-0.977)		<.001	0.602 (0.491-0.713)		.08	0.602 (0.491-0.714)		.08	0.602 (0.524-0.681)		.01
SVM model 4	0.930 (0.872-0.988)		<.001	0.558 (0.450-0.665)		.30	0.663 (0.552-0.774)		.006	0.606 (0.528-0.683)		.009
SVM model 5	0.852 (0.771-0.932)		<.001	0.584 (0.477-0.690)		.13	0.666 (0.556-0.777)		.005	0.620 (0.543-0.697)		.003
SVM model 6	0.769 (0.677-0.862)		<.001	0.619 (0.509-0.729)		.04	0.665 (0.558-0.773)		.004	0.642 (0.565-0.719)		.001
After surgery												
SVM model 7	0.844 (0.763-0.925)		<.001	0.692 (0.588-0.796)		<.001	0.780 (0.687-0.873)		<.001	0.736 (0.666-0.806)		<.001
SVM model 8	0.853 (0.774-0.933)		<.001	0.730 (0.634-0.826)		<.001	0.744 (0.642-0.847)		<.001	0.736 (0.666-0.806)		<.001
SVM model 9	0.753 (0.658-0.847)		<.001	0.677 (0.565-0.789)		.004	0.670 (0.569-0.771)		.002	0.673 (0.598-0.748)		<.001
SVM model 10	0.720 (0.621-0.819)		<.001	0.627 (0.517-0.737)		.03	0.690 (0.583-0.797)		<.001	0.658 (0.581-0.734)		<.001
Preoperative and postoperative markers												
SVM 6+7	0.910 (0.851-0.969)		<.001	0.726 (0.615-0.837)		<.001	0.695 (0.592-0.798)		<.001	0.709 (0.633-0.784)		<.001
SVM 6+8	0.961 (0.917-1.000)		<.001	0.747 (0.654-0.841)		<.001	0.756 (0.655-0.857)		<.001	0.750 (0.682-0.819)		<.001
SVM 6+9	0.952 (0.904-1.000)		<.001	0.700 (0.601-0.798)		<.001	0.731 (0.628-0.834)		<.001	0.714 (0.643-0.785)		<.001
SVM 6+10	0.838 (0.755-0.920)		<.001	0.717 (0.616-0.818)		<.001	0.721 (0.618-0.823)		<.001	0.718 (0.646-0.790)		<.001
SVM 6+TNM^d^	0.847 (0.768-0.927)		<.001	0.684 (0.579-0.788)		.002	0.764 (0.667-0.860)		<.001	0.723 (0.651-0.794)		<.001

a Val: validation.

b AUC: area under the curve.

c SVM: support vector machine.

d TNM: tumor node metastasis.

## Discussion

### Principal Findings

Cancer recurrence remains a major challenge in oncology, significantly impacting patient prognosis. To address this, we developed a machine learning model that predicts recurrence risk, facilitating timely interventions to optimize DFS. Given that surgical and pharmaceutical standards in ESCC treatment generally provide consistent benefits in terms of mortality, DFS is influenced by a combination of multiple factors [15]. Currently, ESCC has a low DFS and imposes a high financial burden on patients, and solely relying on endoscopic follow-up to reduce postoperative recurrence has proven ineffective. In this study, we collected perioperative data from patients with ESCC and conducted follow-ups to develop an artificial intelligence–derived model capable of predicting postoperative recurrence. Implementing this approach is expected to improve DFS. While TNM staging is useful [14], such staging can only be confirmed postoperatively and is only suitable for patients who have already undergone surgery, offering limited value in preoperative planning. Therefore, identifying predictive indicators for DFS preoperatively is important.

Assays for preoperative tumor markers and inflammatory factors [16-20] are cost-effective, convenient, and reliable for diagnosing, treating, and evaluating ESCC prognosis. Surgical factors, such as surgery type, duration of surgery, and intra-operative blood loss, are known risk factors for postoperative recurrence [21-23]; thus, we included these factors into our analysis. In addition, postoperative adjuvant therapy [24] and complications [25] affect prognosis. Collecting comprehensive perioperative data will assist in identifying independent risk factors and facilitate the development of a predictive model for postoperative recurrence. We identified three key findings: (1) Univariate and multivariate Cox regression analyses identified age, ECOG performance status, NLR, CRP, TNM stage, and postoperative complications as independent risk factors for esophageal cancer recurrence. While these factors showed robust predictive value, their combined discriminative ability (AUC=0.676; *P*<.001) was marginally inferior to that of TNM staging alone; (2) The sensitivity of SVM6+8 (combining SVM6 and SVM8, SVM7 excluding complications) for predicting recurrence in patients with ESCC was comparable with that of SVM6+TNM (SVM6 combined with TNM staging) and higher than that of SVM6+TNM. We used a nomogram to input the indexes in the SVM6 into the artificial intelligence program for patients with ESCC who have not yet developed an individualized plan. It can predict and evaluate the postoperative recurrence outcome of patients with ESCC with a sensitivity of 65.73%, specificity of 70.66%, and accuracy of 68.38%. For patients who have undergone surgery, we can enter the indicators in SVM6+TNM into the artificial intelligence program, which can predict and evaluate the postoperative recurrence outcomes of patients with ESCC with sensitivity (81.12%), specificity (71.86%), and accuracy (76.13%); and (3) Survival analysis stratified patients into predicted low-recurrence-risk and high-recurrence-risk groups, based on the SVM model, exhibited significantly prolonged disease-free survival and a markedly lower recurrence rate compared to the predicted high-recurrence-risk group. These findings may contribute to the formulation of personalized follow-up strategies in clinical practice.

### Comparison With Previous Work

Numerous models have been developed to predict the overall survival in postoperative patients with ESCC, but only a few have focused on predicting postoperative recurrence, and their predictive accuracy remains low. Many models, such as logistic regression, decision trees, and random forests, are better suited for large cohort studies. By contrast, the SVM model is suitable for small cohorts that can be independently assigned to 3 groups: 1 test group and 2 verification groups. These groups can be randomly assigned, internally, to assess the practical value of the model. This study was conducted in accordance with the TRIPOD (Transparent Reporting of a Multivariable Prediction Model for Individual Prognosis or Diagnosis) guidelines, which provide guidance for transparently reporting studies that develop, validate, or update diagnostic or prognostic prediction models using clustered data [8]. We developed a model to predict postoperative recurrence based on perioperative data, which we deem essential for improving the overall survival rate of patients with ESCC. Surgical method, duration of surgery, and intraoperative blood loss were not identified as risk factors for postoperative recurrence. Considering the single-center study design and that intraoperative indicators are influenced by the experience and skill level of the surgeon, differences in the duration of surgery and intraoperative blood loss were relatively small, resulting in minimal impact on postoperative recurrence. As the prognostic significance of preoperative blood tests (inflammatory and tumor markers), postoperative pathological stage, and degree of differentiation in patients with ESCC has been previously confirmed [5], we included these indicators into the SVM model. The optimal combination was continuously screened to predict the risk of recurrence of postoperative patients with ESCC to provide guidance for surgical evaluation.

### Strengths and Limitations

Esophagectomy is currently the ideal treatment for patients with ESCC. However, because of the complexity, extensive trauma, and prolonged duration of the surgery, patients experience physiological stress and a high incidence of postoperative complications [24], including anastomotic fistula, pulmonary infection, and respiratory failure. Because of swallowing difficulties and tumor-related metabolic consumption, malnutrition is commonly observed among patients with ESCC. Furthermore, the heightened stress response increases inflammation, weakens the immune system, and impairs tissue repair. Accordingly, reducing surgical risks and improving patient prognosis are crucial. In this study, we analyzed inflammatory indicators and identified NLR, P-CRP, GPS, and CPR as risk factors for postoperative ESCC recurrence [16-20]. Tumor markers are key factors influencing postoperative survival. The results also showed that SCC and CY211 were risk factors for postoperative ESCC recurrence. Additionally, age, ECOG [26], NLR, CPR, TNM stage, and complications were identified as independent risk factors. Our study has the following strengths. First, these prognostic factors were incorporated into an SVM learning model to determine an optimal combination that can be integrated into an artificial intelligence model [27] for a comprehensive evaluation of patient status and prognosis, thereby improving clinical practice. Second, the two validation cohorts further confirmed the model’s accuracy and generalizability. However, this study has some limitations. First, as a retrospective study, it is subject to selection bias. This study’s primary limitation involves potential selection bias from excluding patients lost to follow-up. We addressed this limitation by expanding our sample size, which minimized attrition effects and maintained adequate statistical power for robust conclusions. Second, given the extended follow-up period of this study, new postoperative adjuvant therapies have emerged in clinical practice. Our team has now updated the dataset with recently collected information from esophageal cancer surgery patients, which will enable further in-depth analysis. Finally, the sample size in this study was limited, including only retrospective data from a single health care institution, and randomized validation of the SVM model helps address the limitations of single-center data. However, external validation remains a critical step in ensuring the reliability, generalizability, and clinical applicability of research findings, even in studies with large sample sizes. Despite the advantages of a larger cohort, issues such as overfitting, selection bias, or dataset-specific artifacts may still arise. Thus, to further enhance its clinical usability, we plan to implement this predictive model across multiple hospitals.

### Future Directions

In this study, we used the SVM model and analyzed the ROC curve to qualitatively and quantitatively evaluate the predictive ability of the model. In addition, a nomogram was generated to evaluate the DFS of patients with ESCC. Subsequently, treatment plans were adopted based on the predicted high- and low-risk of recurrence. Differences between the high- and low-risk groups guided individualized medical treatments, such as personalized surgical planning (or appropriate surgical procedures), optimization of radiotherapy and chemotherapy dosage and timing, and selection of appropriate follow-up intervals. Patients in the high-risk group for postoperative recurrence should undergo enhanced follow-up with close monitoring through gastroscopy, histopathological examination, and imaging studies. By contrast, follow-up schedules for the low-risk group should be based on blood test results to ensure appropriate monitoring. The development of this artificial intelligence model enables early prediction of postoperative recurrence risk in patients with ESCC while facilitating the generation of personalized medical plans, such as optimized postoperative radiotherapy and chemotherapy regimens as well as reasonable follow-up schedules [28]. By reducing unnecessary postoperative examinations, this model enhances the efficiency of follow-up care. It is particularly well-suited for use in towns and community health care settings to assist local medical practitioners in accurately assessing patient status, reducing the rate of recurrence of postoperative ESCC, and improving the 5-year survival rate.

### Conclusion

Age, ECOG performance status, NLR, CPR, TNM, and complications were identified as independent risk factors for postoperative ESCC recurrence. These factors, which affect patient prognosis, were incorporated into the SVM learning model to determine the optimal risk-predictive combination. This model, integrated with an artificial intelligence model, provides a comprehensive assessment of patient status and prognosis, assisting the development of follow-up treatment plans.

## Supplementary material

10.2196/68027 Multimedia Appendix 1 Baseline characteristics of the participants.

10.2196/68027 Multimedia Appendix 2 Quantitative correlation analysis between preoperative tumor markers and postoperative clinical indicators in patients with esophageal squamous cell carcinoma (ESCC).

10.2196/68027 Multimedia Appendix 3 Disease-free survival (DFS) rates at 1, 3, and 5 years following esophagectomy.

10.2196/68027 Multimedia Appendix 4 Quantitative evaluation of the precise diagnosis of esophagus cancer with any 3 or more indexes by the support vector machine (SVM) model.

10.2196/68027 Multimedia Appendix 5 A heatmap showing the high- and low-distribution profiles of risk factors affecting recurrence in patients with esophageal squamous cell carcinoma (ESCC).

10.2196/68027 Multimedia Appendix 6 A nomogram was developed using the available data to predict disease-free survival (SVM 6).

10.2196/68027 Multimedia Appendix 7 The findings indicated a high degree of uniformity between the predicted and observed probabilities of survival in the training set and internal validation set (SVM 6).

## Abbreviations:

AUC: area under the curve
CPR: C-reactive protein-to-prealbumin ratio
CRP: C-reactive protein
CY211: cytokeratin 19 fragment
DFS: disease-free survival
ECOG: Eastern Cooperative Oncology Group
ESCC: esophageal squamous cell carcinoma
GPS: Glasgow prognostic score
HR: hazard ratio
LMR: Lymphocyte to monocyte ratio
NLR: neutrophil-to-lymphocyte ratio
P-CRP: the platelet × C-reactive protein multiplier value
PHR: predicted high-risk group
ROC: receiver operating characteristic
SCC: squamous cell carcinoma antigen
SVM: support vector machine
TNM: tumor node metastasis
