# Supplementary material for: Predicting Postoperative Recurrence Using a Support Vector Machine for Patients With Esophageal Squamous Cell Carcinoma: Machine Learning Modeling Development and Validation Study
Source: JMIR Cancer. 2025 Oct 23;11:e68027. doi: 10.2196/68027 (PMC12548966; doi:10.2196/68027)
Supplement: Multimedia Appendix 1 [file cancer-v11-e68027-s001.docx]

| **Table S1 Baseline characteristics of the participants.** | | | | | | |
| --- | --- | --- | --- | --- | --- | --- |
| Clinical Indicators | Patients Cohort | Total n(%) | Recurrence group n(%) | Non-recurrence group n(%) | χ2 | P value |
| Age (years) | <66 | 154 | 80 | 74 | 3.911 | 0.048 |
|  | ≥66 | 157 | 64 | 93 |  |  |
| Gender | Male | 241 | 113 | 128 | 0.148 | 0.701 |
|  | Female | 70 | 31 | 39 |  |  |
| ECOG | <1 | 142 | 45 | 97 | 22.44 | <0.001 |
|  | ≥1 | 169 | 99 | 70 |  |  |
| NLR | <2.43 | 154 | 57 | 97 | 10.587 | 0.001 |
|  | ≥2.43 | 157 | 87 | 70 |  |  |
| LMR | <4.28 | 155 | 76 | 79 | 0.926 | 0.336 |
|  | ≥4.28 | 156 | 68 | 88 |  |  |
| P-CRP | <152.40 | 154 | 65 | 89 | 2.057 | 0.152 |
|  | ≥152.40 | 157 | 79 | 78 |  |  |
| GPS | <1 | 153 | 64 | 89 | 2.423 | 0.12 |
|  | ≥1 | 158 | 80 | 78 |  |  |
| CRP(mg/dL) | <0.90 | 152 | 67 | 85 | 0.591 | 0.442 |
|  | ≥0.90 | 159 | 77 | 82 |  |  |
| CPR | <0.0037 | 157 | 59 | 98 | 9.702 | 0.002 |
|  | ≥0.0037 | 154 | 85 | 69 |  |  |
| SCC(ng/ml) | <0.90 | 143 | 58 | 85 | 3.511 | 0.061 |
|  | ≥0.90 | 168 | 86 | 82 |  |  |
| CY211(ng/ml) | <2.65 | 154 | 56 | 98 | 11.744 | 0.001 |
|  | ≥2.65 | 156 | 87 | 69 |  |  |
| Surgical Method | Mckeown | 206 | 95 | 111 | 0.008 | 0.927 |
|  | Sweet | 105 | 49 | 56 |  |  |
| Intraoperative blood loss | <200 | 88 | 37 | 51 | 0.757 | 0.384 |
|  | ≥200 | 221 | 105 | 116 |  |  |
| Operative Time | <210 | 145 | 72 | 73 | 1.391 | 0.238 |
|  | ≥210 | 163 | 70 | 93 |  |  |
| Tumor Location | Upper | 17 | 8 | 9 | 0.395 | 0.821 |
|  | Middle | 217 | 98 | 119 |  |  |
|  | Lower | 75 | 37 | 38 |  |  |
| Tumor Size | <3.0 | 109 | 39 | 70 | 7.473 | 0.006 |
|  | ≥3.0 | 202 | 105 | 97 |  |  |
| T | T1+2 | 148 | 55 | 93 | 9.488 | 0.002 |
|  | T3+4 | 163 | 89 | 74 |  |  |
| N | N0 | 173 | 51 | 122 | 44.376 | <0.001 |
|  | N1+2+3 | 138 | 93 | 45 |  |  |
| TNM | I+II | 178 | 55 | 123 | 39.719 | <0.001 |
|  | III+IV | 133 | 89 | 44 |  |  |
| Cell Differentiation | Well+Moderate | 221 | 91 | 130 | 8.07 | 0.005 |
|  | Poorly | 90 | 53 | 37 |  |  |
| Adjuvant therapy | 0 | 170 | 58 | 112 | 22.39 | <0.001 |
|  | 1 | 141 | 86 | 55 |  |  |
| Complications | 0 | 189 | 80 | 109 | 3.061 | 0.08 |
|  | 1 | 122 | 64 | 58 |  |  |
