# Supplementary material for: Predicting Postoperative Recurrence Using a Support Vector Machine for Patients With Esophageal Squamous Cell Carcinoma: Machine Learning Modeling Development and Validation Study
Source: JMIR Cancer. 2025 Oct 23;11:e68027. doi: 10.2196/68027 (PMC12548966; doi:10.2196/68027)
Supplement: Multimedia Appendix 2 [file cancer-v11-e68027-s002.docx]

| **Table S2 Quantitative correlation analysis between preoperative tumor markers and postoperative clinical indicators in patients with ESCC.** | | | | | | | | | | | | | | | | | | | | | | |
| --- | --- | --- | --- | --- | --- | --- | --- | --- | --- | --- | --- | --- | --- | --- | --- | --- | --- | --- | --- | --- | --- | --- |
| Markers | Gender | Age | ECOG | NLR | SCC | CY211 | CRP | GPS | LMR | PCRP | CPR | Tumor Location | Complications | T | N | TNM | Cell Differentiation | Tumor Size | Surgical Method | Operative Time | Intraoperative blood loss |  |
| Gender | 1 |  |  |  |  |  |  |  |  |  |  |  |  |  |  |  |  |  |  |  |  |  |
| Age | .171^b^ | 1 |  |  |  |  |  |  |  |  |  |  |  |  |  |  |  |  |  |  |  |  |
| ECOG | -0.095 | 0.190^b^ | 1 |  |  |  |  |  |  |  |  |  |  |  |  |  |  |  |  |  |  |  |
| NLR | -0.135^a^ | -0.015 | 0.342^b^ | 1 |  |  |  |  |  |  |  |  |  |  |  |  |  |  |  |  |  |  |
| SCC | -0.057 | 0.017 | 0.151^b^ | 0.146^a^ | 1 |  |  |  |  |  |  |  |  |  |  |  |  |  |  |  |  |  |
| CY211 | -0.028 | 0.167^b^ | 0.108 | 0.085 | 0.092 | 1 |  |  |  |  |  |  |  |  |  |  |  |  |  |  |  |  |
| CRP | -0.032 | -0.065 | 0.111 | 0.142^a^ | 0.082 | 0.160^b^ | 1 |  |  |  |  |  |  |  |  |  |  |  |  |  |  |  |
| GPS | -0.031 | -0.018 | 0.173^b^ | 0.261^b^ | 0.109 | 0.240^b^ | 0.301^b^ | 1 |  |  |  |  |  |  |  |  |  |  |  |  |  |  |
| LMR | .189^b^ | 0.047 | -0.108 | -0.257^b^ | -0.172^b^ | -0.134^a^ | -0.095 | -0.128^a^ | 1 |  |  |  |  |  |  |  |  |  |  |  |  |  |
| PCRP | -0.041 | -0.060 | 0.108 | 0.114^a^ | 0.095 | 0.153^b^ | 0.956^b^ | 0.248^b^ | -0.069 | 1 |  |  |  |  |  |  |  |  |  |  |  |  |
| CPR | -0.037 | -0.045 | 0.137^a^ | 0.144^a^ | 0.080 | 0.136^a^ | 0.941^b^ | 0.270^b^ | -0.100 | 0.876^b^ | 1 |  |  |  |  |  |  |  |  |  |  |  |
| Tumor Location | -0.244^b^ | -0.050 | 0.078 | 0.129^a^ | 0.118^a^ | 0.114^a^ | 0.057 | 0.025 | -0.065 | 0.044 | 0.067 | 1 |  |  |  |  |  |  |  |  |  |  |
| Complications | 0.009 | 0.089 | 0.203^b^ | -0.052 | -0.113^a^ | 0.031 | -0.059 | -0.062 | -0.130^a^ | -0.036 | -0.086 | -0.100 | 1 |  |  |  |  |  |  |  |  |  |
| T | -0.147^b^ | -0.039 | 0.140^a^ | 0.232^b^ | 0.237^b^ | 0.096 | 0.144^a^ | 0.117^a^ | -0.162^b^ | 0.127^a^ | 0.176^b^ | 0.160^b^ | -0.064 | 1 |  |  |  |  |  |  |  |  |
| N | 0.004 | -0.070 | 0.161^b^ | 0.245^b^ | 0.284^b^ | -0.032 | 0.127^a^ | 0.089 | -0.069 | 0.111 | 0.126^a^ | 0.095 | -0.106 | 0.387^b^ | 1 |  |  |  |  |  |  |  |
| TNM | -0.015 | -0.072 | 0.170^b^ | 0.251^b^ | 0.257^b^ | 0.056 | 0.116^a^ | 0.082 | -0.101 | 0.102 | 0.122^a^ | 0.144^a^ | -0.046 | 0.688^b^ | 0.831^b^ | 1 |  |  |  |  |  |  |
| Cell Differentiation | -0.010 | 0.088 | 0.228^b^ | 0.217^b^ | 0.046 | -0.095 | 0.158^b^ | 0.145^a^ | 0.075 | 0.165^b^ | 0.145^a^ | -0.050 | 0.095 | 0.276^b^ | 0.421^b^ | 0.461^b^ | 1 |  |  |  |  |  |
| Tumor Size | -0.025 | 0.076 | 0.112^a^ | 0.184^b^ | 0.207^b^ | 0.172^b^ | 0.206^b^ | 0.170^b^ | -0.083 | 0.179^b^ | 0.241^b^ | 0.128^a^ | -0.142^a^ | 0.470^b^ | 0.267^b^ | 0.355^b^ | 0.089 | 1 |  |  |  |  |
| Surgical Method | -0.108 | -0.039 | -0.129^a^ | -0.019 | -0.091 | 0.039 | 0.127^a^ | 0.102 | 0.197^b^ | 0.121^a^ | 0.129^a^ | 0.153^b^ | -0.239^b^ | -0.093 | -0.138^a^ | -0.141^a^ | -0.024 | 0.146^b^ | 1 |  |  |  |
| Operative Time | -0.079 | 0.036 | 0.094 | 0.056 | -0.049 | -0.141^a^ | -0.145^a^ | -0.024 | -0.050 | -0.126^a^ | -0.127^a^ | 0.079 | 0.235^b^ | -0.033 | 0.063 | 0.003 | 0.035 | 0.103 | -0.266^b^ | 1 |  |  |
| Intraoperative blood loss | -0.134^a^ | -0.021 | 0.000 | -0.011 | -0.062 | 0.079 | -0.046 | 0.120^a^ | 0.001 | -0.048 | -0.014 | 0.025 | 0.157^b^ | 0.099 | 0.028 | 0.042 | 0.038 | 0.125^a^ | 0.011 | 0.426^b^ | 1 |  |
| a. Correlation is significant at the 0.05 level (2-tailed). b. Correlation is significant at the 0.01 level (2-tailed). T=Invasion depth ; N =Lymph node metastasis; TNM=TNM staging; | | | | | | | | | | | | | | | | | | | | | | |
