# Supplementary figures and images for: Predicting Postoperative Recurrence Using a Support Vector Machine for Patients With Esophageal Squamous Cell Carcinoma: Machine Learning Modeling Development and Validation Study
Source: JMIR Cancer. 2025 Oct 23;11:e68027. doi: 10.2196/68027 (PMC12548966; doi:10.2196/68027)

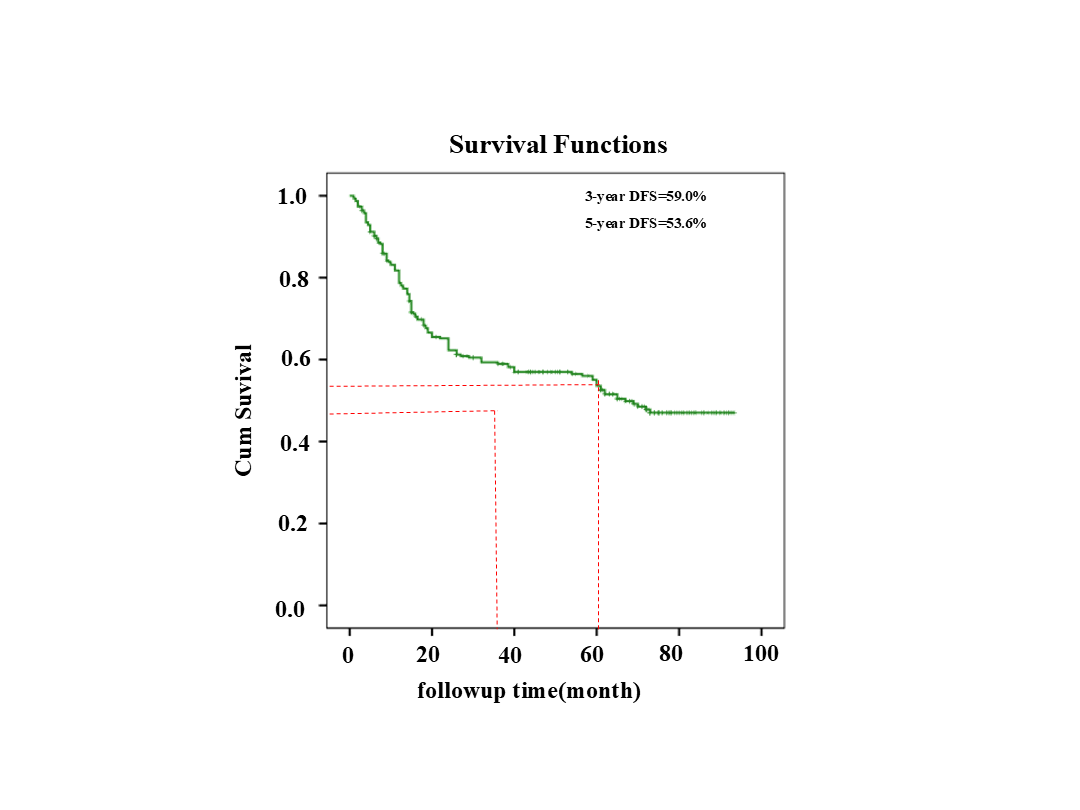

Supplement: Multimedia Appendix 3 [file cancer-v11-e68027-s003.png]

Fig S2

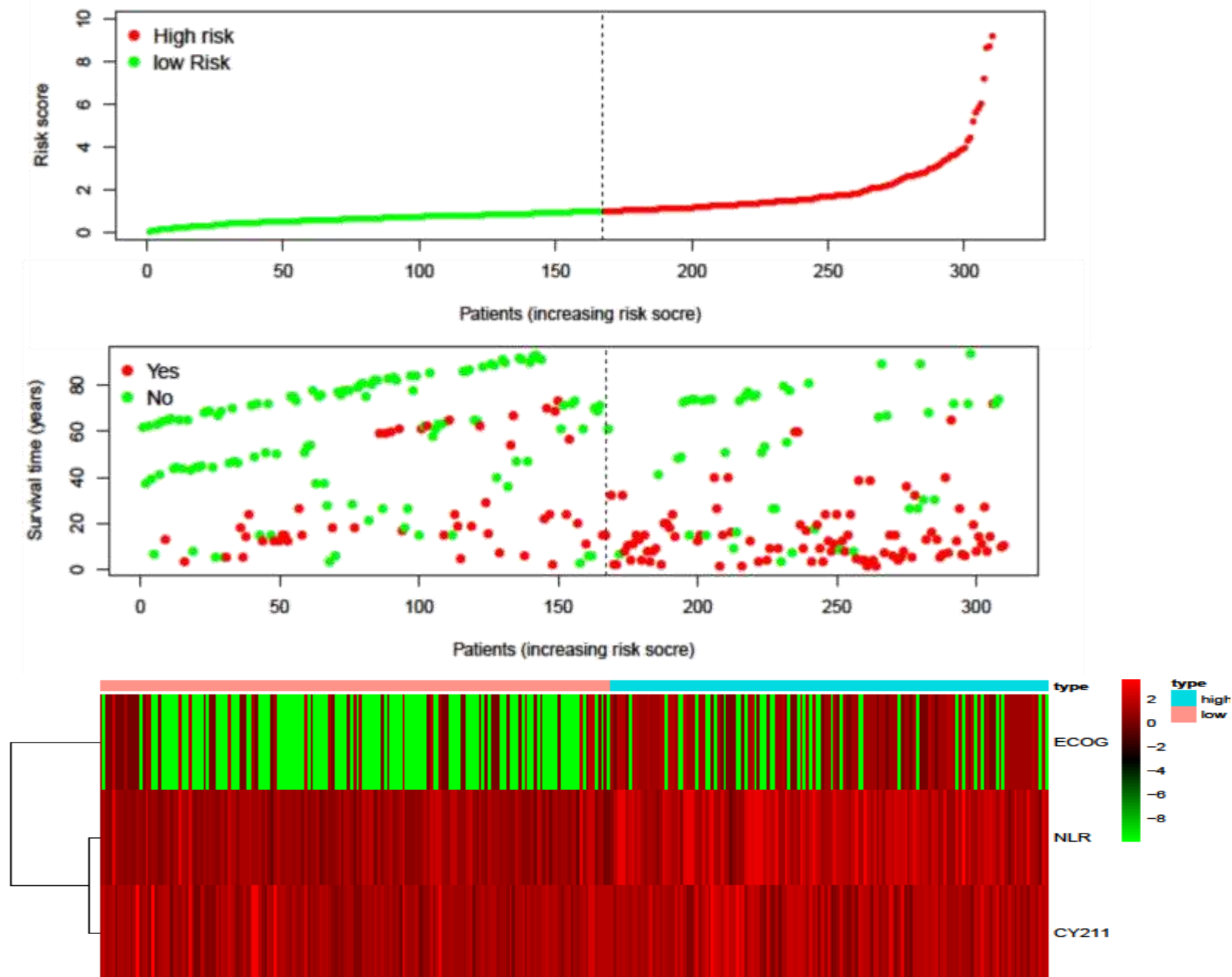

Supplement: Multimedia Appendix 5 [file cancer-v11-e68027-s005.pdf]

Points

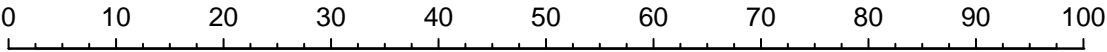

ECOG

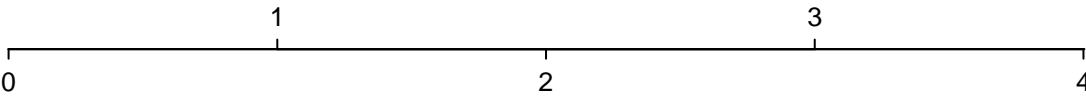

CY211

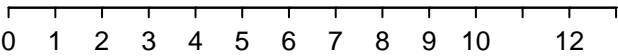

NLR

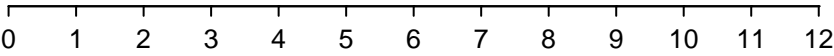

Total Points

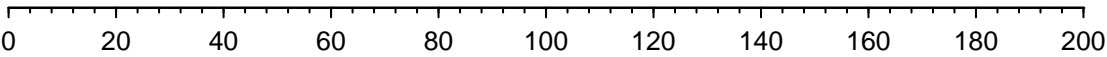

Linear Predictor

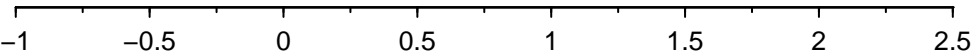

1-year DFS

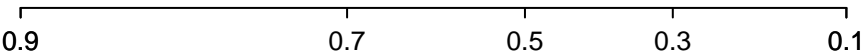

3-year DFS

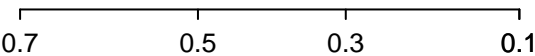

5-year DFS

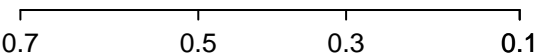

Supplement: Multimedia Appendix 6 [file cancer-v11-e68027-s006.pdf]

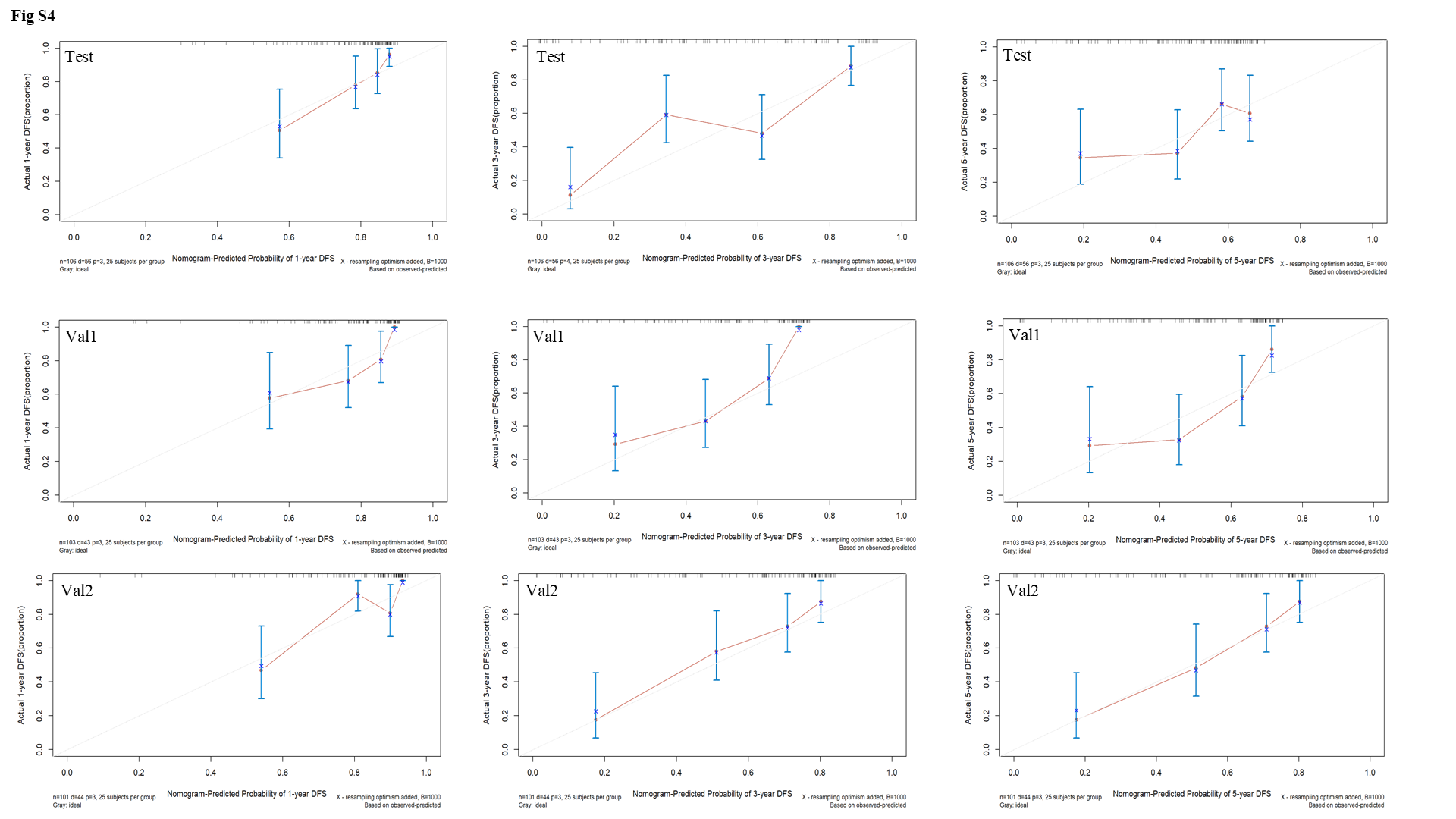

Supplement: Multimedia Appendix 7 [file cancer-v11-e68027-s007.png]
