# Supplementary material for: Predicting Postoperative Recurrence Using a Support Vector Machine for Patients With Esophageal Squamous Cell Carcinoma: Machine Learning Modeling Development and Validation Study
Source: JMIR Cancer. 2025 Oct 23;11:e68027. doi: 10.2196/68027 (PMC12548966; doi:10.2196/68027)
Supplement: Multimedia Appendix 4 [file cancer-v11-e68027-s004.docx]

| **Table S3. Quantitative evaluation of the precise diagnosis of Esophagus cancer with any three or more indexes by SVM model** | | | | | | | | | | | | | | | | | | | | | | | |
| --- | --- | --- | --- | --- | --- | --- | --- | --- | --- | --- | --- | --- | --- | --- | --- | --- | --- | --- | --- | --- | --- | --- | --- |
| Variable combinations | Sensitivity(%) | | |  | Specificity(%) | | |  | Yuedens' Index(%) | | |  | PPV(%) | | |  | NPV(%) | | |  | Accuracy(%) | | |
|  | Test | Val 1 | Val 2 |  | Test | Val 1 | Val 2 |  | Test | Val 1 | Val 2 |  | Test | Val 1 | Val 2 |  | Test | Val 1 | Val 2 |  | Test | Val 1 | Val 2 |
| **Before Surgery** | |  |  |  |  |  |  |  |  |  |  |  |  |  |  |  |  |  |  |  |  |  |  |
| SVM 1 | 94.12 | 66.67 | 60.98 |  | 98.21 | 63.33 | 54.90 |  | 92.33 | 30.00 | 15.88 |  | 97.96 | 60.71 | 52.08 |  | 94.83 | 69.09 | 63.64 |  | 96.26 | 64.86 | 57.61 |
| SVM 2 | 94.12 | 70.59 | 60.98 |  | 98.21 | 63.33 | 56.86 |  | 92.33 | 33.92 | 17.84 |  | 97.96 | 62.07 | 53.19 |  | 94.83 | 71.70 | 64.44 |  | 96.26 | 66.67 | 58.70 |
| SVM 3 | 89.29 | 60.47 | 59.09 |  | 94.00 | 60.00 | 61.40 |  | 83.29 | 20.47 | 20.49 |  | 94.34 | 52.00 | 54.17 |  | 88.68 | 67.92 | 66.04 |  | 91.51 | 60.19 | 60.40 |
| SVM 4 | 86.00 | 59.18 | 65.91 |  | 100.00 | 52.38 | 66.67 |  | 86.00 | 11.56 | 32.58 |  | 100.00 | 49.15 | 63.04 |  | 88.33 | 62.26 | 69.39 |  | 93.20 | 55.36 | 66.32 |
| SVM 5 | 76.00 | 61.22 | 56.82 |  | 94.34 | 55.56 | 76.47 |  | 70.34 | 16.78 | 33.29 |  | 92.68 | 51.72 | 67.57 |  | 80.65 | 64.81 | 67.24 |  | 85.44 | 58.04 | 67.37 |
| SVM 6 | 67.86 | 60.47 | 68.18 |  | 86.00 | 63.33 | 64.91 |  | 53.86 | 23.80 | 33.09 |  | 84.44 | 54.17 | 60.00 |  | 70.49 | 69.09 | 72.55 |  | 76.42 | 62.14 | 66.34 |
| **After Surgery** | |  |  |  |  |  |  |  |  |  |  |  |  |  |  |  |  |  |  |  |  |  |  |
| SVM Model 7 | 92.86 | 76.74 | 84.09 |  | 76.00 | 61.67 | 71.93 |  | 68.86 | 38.41 | 56.02 |  | 81.25 | 58.93 | 69.81 |  | 90.48 | 78.72 | 85.42 |  | 84.91 | 67.96 | 77.23 |
| SVM Model 8 | 82.00 | 71.43 | 70.45 |  | 88.68 | 74.60 | 78.43 |  | 70.68 | 46.03 | 48.89 |  | 87.23 | 68.63 | 73.81 |  | 83.93 | 77.05 | 75.47 |  | 85.44 | 73.21 | 74.74 |
| SVM Model 9 | 81.82 | 63.64 | 67.27 |  | 68.75 | 71.74 | 66.67 |  | 50.57 | 35.38 | 33.94 |  | 64.29 | 68.29 | 66.07 |  | 84.62 | 67.35 | 67.86 |  | 74.07 | 67.78 | 66.96 |
| SVM Model 10 | 66.07 | 60.47 | 59.09 |  | 78.00 | 65.00 | 78.95 |  | 44.07 | 25.47 | 38.04 |  | 77.08 | 55.32 | 68.42 |  | 67.24 | 69.64 | 71.43 |  | 71.70 | 63.11 | 70.30 |
| **All markers** | |  |  |  |  |  |  |  |  |  |  |  |  |  |  |  |  |  |  |  |  |  |  |
| SVM 6+7 | 91.07 | 72.50 | 72.34 |  | 90.91 | 72.73 | 66.67 |  | 81.98 | 45.23 | 39.01 |  | 89.47 | 70.73 | 64.15 |  | 92.31 | 74.42 | 74.51 |  | 90.98 | 72.62 | 69.23 |
| SVM 6+8 | 94.00 | 79.59 | 72.73 |  | 98.11 | 69.84 | 78.43 |  | 92.11 | 49.43 | 51.16 |  | 97.92 | 67.24 | 74.42 |  | 94.55 | 81.48 | 76.92 |  | 96.12 | 74.11 | 75.79 |
| SVM 6+9 | 96.00 | 79.59 | 79.55 |  | 94.34 | 60.32 | 66.67 |  | 90.34 | 39.91 | 46.21 |  | 94.12 | 60.94 | 67.31 |  | 96.15 | 79.17 | 79.07 |  | 95.15 | 68.75 | 72.63 |
| SVM 6+10 | 87.50 | 76.74 | 70.45 |  | 80.00 | 66.67 | 73.68 |  | 67.50 | 43.41 | 44.14 |  | 83.05 | 62.26 | 67.39 |  | 85.11 | 80.00 | 76.36 |  | 83.96 | 70.87 | 72.28 |
| SVM 6+TNM | 87.50 | 76.74 | 77.27 |  | 82.00 | 60.00 | 75.44 |  | 69.50 | 36.74 | 52.71 |  | 84.48 | 57.89 | 70.83 |  | 85.42 | 78.26 | 81.13 |  | 84.91 | 66.99 | 76.24 |
| Note: test group, validation group 1, validation group 2 were randomly divided into three independent SVM model groups. The test group was also known as the learning group, then using two validation groups to recognite the precise of the diagnosis. The closer the sensitivity and specificity of the test group, the higher the accuracy of the diagnostic model. Data are presented as percentages. PPV= positive predictive value, NPV= negative predictive value. Val = Validation. SVM 1=All markers before surgery(ECOG,NLR,CPR,CY211,SCC,P-CRP,GPS,age); SVM 2=SVM Model 1(omit P-CRP); SVM 3=SVM Model 2(omit GPS); SVM 4= SVM Model 3(omit SCC); SVM 5=SVM Model 4(omit age); SVM 6=SVM Model 5(omit CPR): ECOG, NLR, CY211; SVM 7=TNM, Adjuvant therapy, Diff, Size, Complications. SVM8=SVM7(omit Complications), SVM9=SVM8(omit Size), SVM10=SVM9(omit ​Diff). | | | | | | | | | | | | | | | | | | | | | | | |
